# Supplementary material for: A steep switching WSe2 impact ionization field-effect transistor
Source: Nat Commun. 2022 Oct 14;13:6076. doi: 10.1038/s41467-022-33770-3 (PMC9568662; doi:10.1038/s41467-022-33770-3)
Supplement: Supplementary file 1 — Supplementary Information [file 41467_2022_33770_MOESM1_ESM.pdf]

Supporting Information

A steep switching WSe_2 impact ionization field-effect transistor

Haeju Choi[1], Jinshu Li[1], Taeho Kang[1], Chanwoo Kang[1], Hyeonje Son[1], Jongwook Jeon[2], Euyheon Hwang[1,3], and Sungjoo Lee[1,3*]*

[1]SKKU Advanced Institute of Nanotechnology (SAINT), Sungkyunkwan University, Suwon 440-746, Korea

[2]Department of Electrical and Electronics Engineering, Konkuk University, Seoul, 05029, Republic of Korea

[3]Department of Nano Engineering, Sungkyunkwan University, Suwon 440-746, Korea

E-mail: leesj@skku.edu; heh8232@gmail.com

Contents

Supplementary Note 1. Device fabrication

a. Fabrication process

b. Optimal microscopy, scanning electron microscopy (SEM), and transmission electron microscopy (TEM) images of WSe_2 flakes and devices

Supplementary Note 2. Impact ionization properties of WSe_2

a. Band structure of WSe_2

b. Impact ionization characteristics for various lengths, thicknesses, and temperatures

c. Gate bias effect on impact ionization

d. Output characteristics during V_{DS} sweeps

e. Estimation of ionization coefficient (lucky drift mechanism)

f. Relationship between the multiplication factor and channel length

Supplementary Note 3. Properties and control of the WSe_2 I^2FET

a. Electrical properties of the WSe_2 I^2FET

b. Control of operating voltages via gated and ungated region length modulation

c. Negligible hysteresis characteristics of the impact ionization process

d. Gate leakage current

e. Reliability of the impact ionization phenomenon

Supplementary Note 4. Complementary inverter with WSe_2 I^2FET

a. Noise margins of WSe_2 I^2FET inverter

b. Scalable voltage drop of WSe_2 I^2FET inverter

Supplementary Note 1. Device fabrication

a. Fabrication process

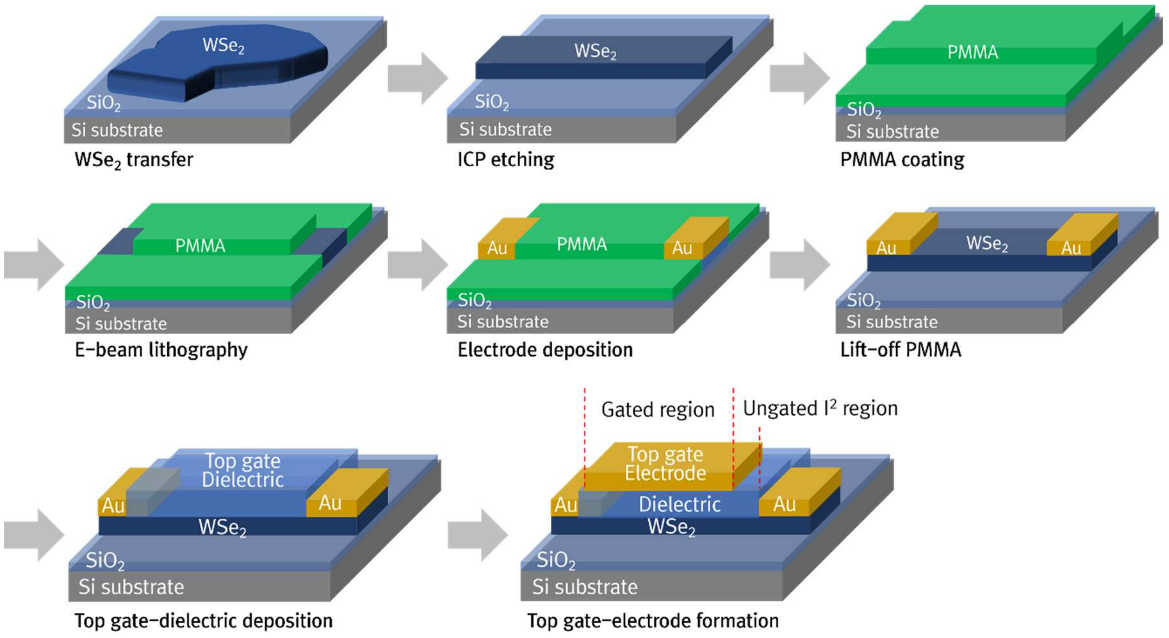

Supplementary Figure 1. Schematic illustrations of the process flow for WSe_2 I^2FET fabrication.

Supplementary Figure 1 illustrates the WSe_2 FET and WSe_2 I^2FETs fabrication processes. To prepare the WSe_2 channel layers, WSe_2 flakes were transferred from a bulk WSe_2 crystal onto a SiO_2/Si substrate using a mechanical exfoliation method. The flakes were made into elongated squares using an inductively coupled plasma etching process. On the WSe_2 channel, we spin-coated double-electron resistor layers with polymethyl methacrylate at 450 K and 2000 rpm for 5 s and at 950 K and 4000 rpm for 35 s. Each layer was baked at 180 °C for 2 min on a hot plate. After the source and drain electrode patterns were defined using electron beam lithography, Au (approximately 25 nm thick) was deposited using an electron beam evaporator for source and drain electrode formation.

For the WSe_2 I^2FET fabrication, we repeated this e-beam lithography and e-beam deposition process two more times to deposit a top-gate dielectric layer (SiO_2 approximately 20 nm thick) and a top gate electrode (Au approximately 80 nm thick). The top-gate electrode was deposited locally closer to the source side such that a homogeneous WSe_2 junction could be formed between the gated and ungated WSe_2 channels (drain side), where impact ionization occurs under an electric field higher than E_{CR}. The fabricated devices were annealed at 200 °C in an Ar atmosphere for 2 h to eliminate residues on their surfaces.

b. Optimal microscopy, scanning electron microscopy (SEM), and transmission electron microscopy (TEM) images of WSe_2 flakes and devices

Supplementary Figure 2 presents optical images of (a) exfoliated WSe_2 flakes, (b) the I_{CP}-etched WSe_2 channel, (c) fabricated WSe_2 FET, and (d) fabricated WSe_2 I^2FET. The candidate flakes of WSe_2 were selected based on color contrast, and their detailed thickness characterization was conducted using atomic force microscopy. Multilayer flakes with a thickness of approximately 50 nm were selected. The length information of the representative WSe_2 I^2FET is presented in parts (e) and (f). The ungated region length is approximately 300 nm, as shown in the (e) SEM image. A cross-sectional TEM image is presented in part (f), which helps us verify that the stack is clean without any contamination following the device fabrication processes.

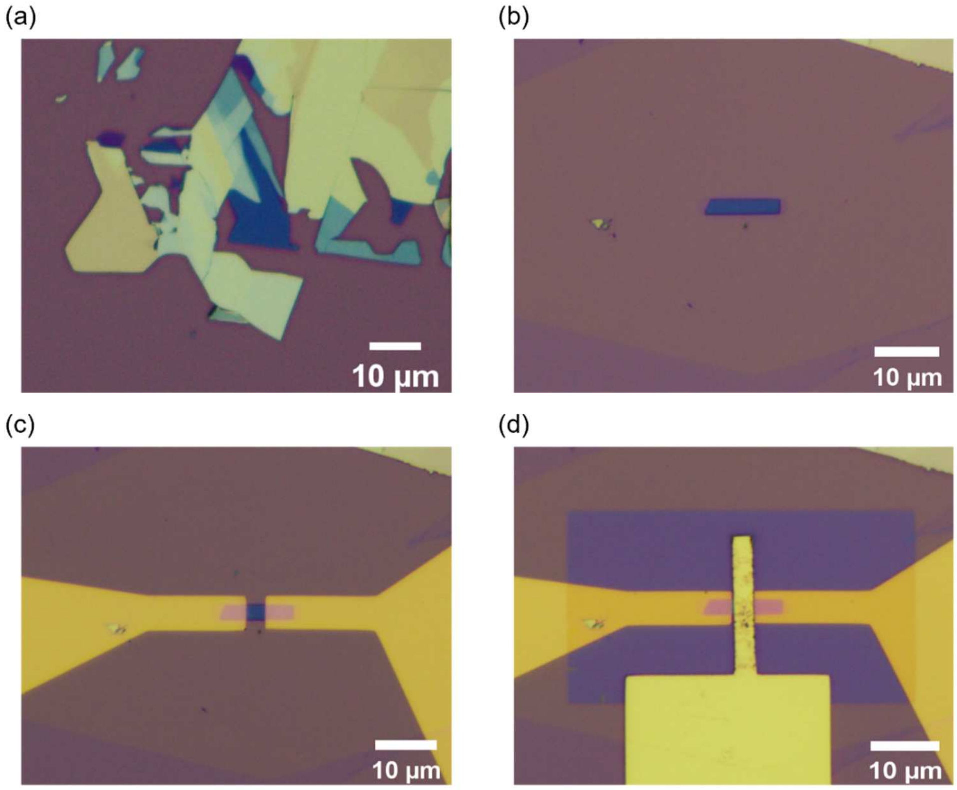

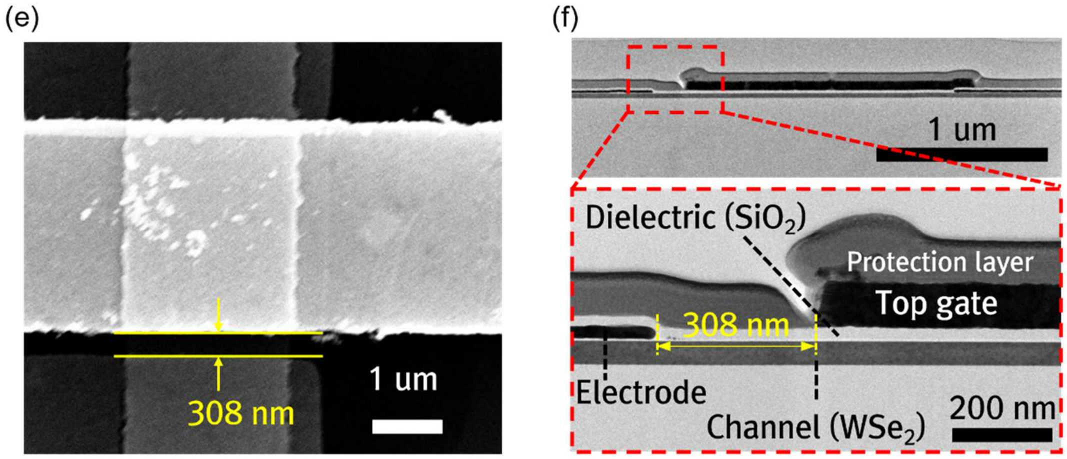

Supplementary Figure 2. Optical images of (a) exfoliated WSe_2 flakes, (b) I_{CP}-etched multilayer flake, fabricated (c) FET, and (d) I^2FET. (e) Surface SEM image and (f) cross-sectional TEM image of the WSe_2 I^2FET.

Supplementary Note 2. Impact ionization properties of WSe_2

a. Band structure of WSe_2

Structural optimization and band structure calculations were conducted using the Quantum Espresso Package [1]. We adopted the Perdew-Burke-Ernzerhof generalized gradient approximation for the exchange-correlation function, an ultrasoft pseudopotential, and energy cutoff of 70 Ry with 8 ×8 ×2 k-points for the supercell. For the Van der Waals forces, the semi-empirical Grimme's DFT-D3 van der Waals correction [2] was applied.

Supplementary Figure 3 presents the band structure of multilayer WSe_2 in the momentum space along the line Γ-M-K-Γ. The effective mass was calculated using a parabolic approximation with a unit of m_0, which is the mass of an electron and is presented near each conduction valley. The effective hole mass is negative.

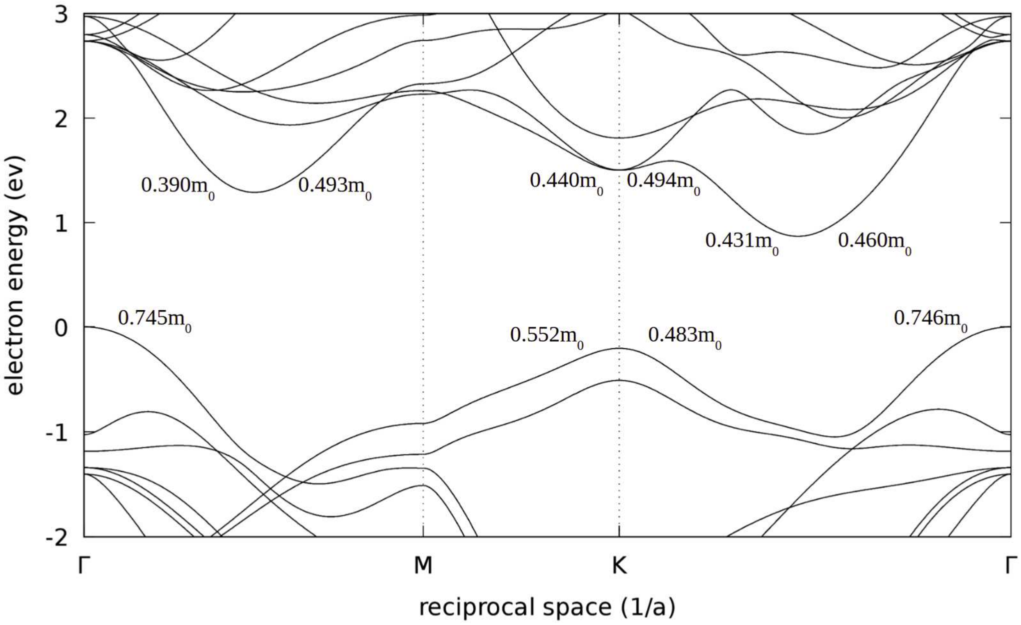

Supplementary Figure 3. DFT-based band structures for multilayer WSe_2.

b. Impact ionization characteristics for various lengths, thicknesses, and temperatures

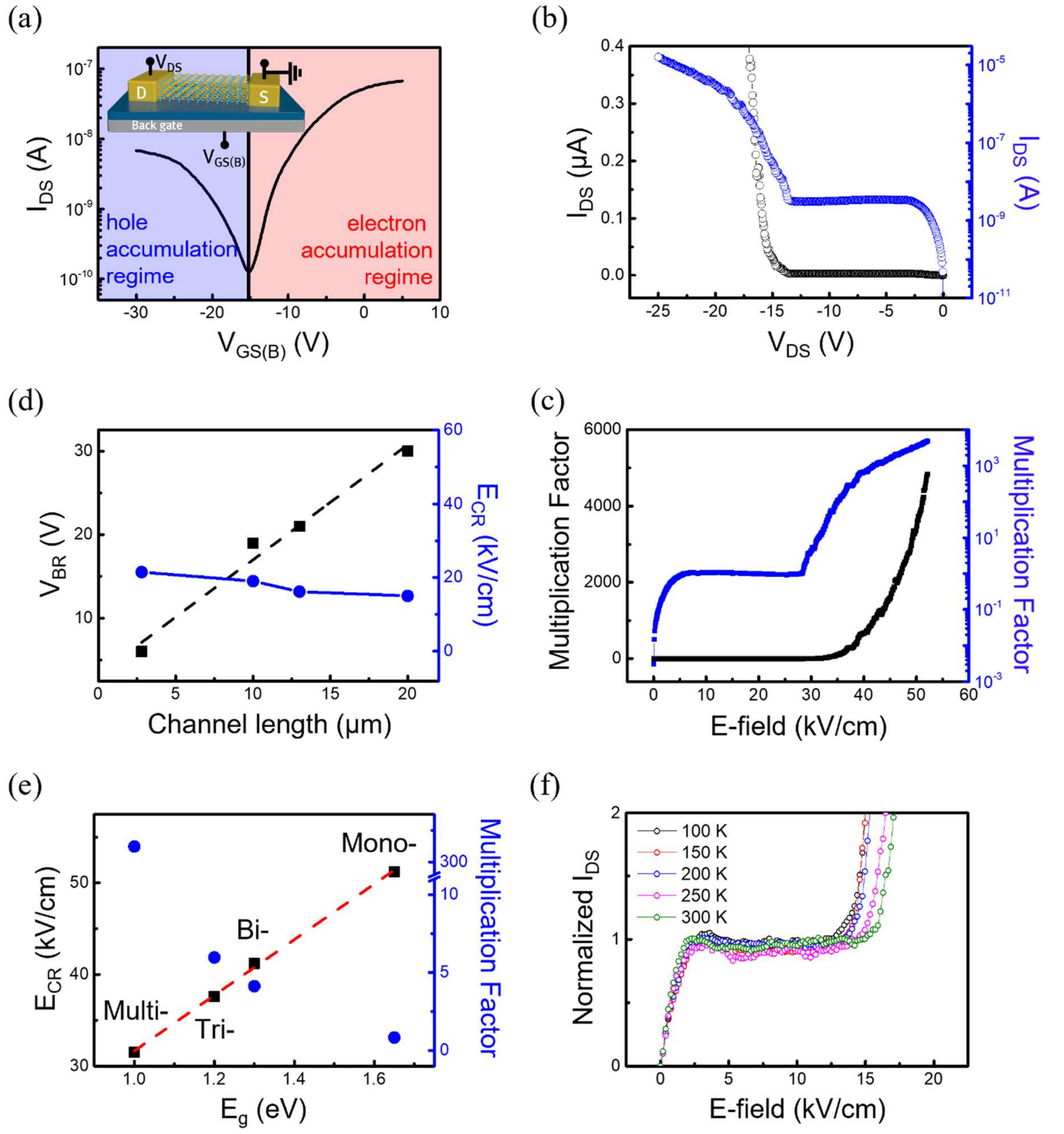

Supplementary Figure 4. (a) Transfer curve and (b) output curve exhibiting impact ionization in the high-V_{DS} regime (V_{DS} < −13.8 V). I_{DS} increases linearly in the low-E-field regime, whereas it increases abruptly in the high-E-field regime (log scale and linear scale plotted with blue and black lines, respectively). The inset shows the schematic of the WSe_2 FET. (c) Calculated multiplication factor (M) as a function of the electric field. (d) Calculated V_{BR} and E_{CR} of WSe_2 FETs with various channel lengths. (e) E_{CR} values and multiplication factors in the same electric field (E = 52 kV/cm) with different energy band gaps varying with the number

of layers. (f) Normalized I_{DS} as a function of electric field for various temperatures measured at $V_{GS(B)} = -30$ V.

We fabricated a simple WSe_2 FET in which carrier doping in the channel was modulated only by the back gate (see the inset in Supplementary Fig. 4a; the fabrication process is shown in Supplementary Fig. 1). Supplementary Figure 4a shows the transfer curve of the WSe_2 FET (channel length: ~4.8 μm), which has typical ambipolar transport characteristics and exhibits hole doping for $V_{GS(B)} < -15$ V and electron doping for $V_{GS(B)} > -15$ V. Supplementary Figure 4b shows the hole current I_{DS} as a function of drain voltage V_{DS} at a fixed back-gate voltage of $V_{GS(B)} = -25$ V. The black (blue) line indicates the measured current on a linear (semilogarithmic) scale. At low drain voltages, the current increased with drain voltage and approached saturation values at an intermediate voltage. However, as the voltage increased further (i.e., $V_{DS} < -13.8$ V), an abrupt increase in the current was observed, and breakdown occurred at breakdown voltage V_{BR}, which was attributed to the impact ionization process. The multiplication factor (M) extracted from the measured I_{DS} is presented in Supplementary Fig. 4c as a function of the electric field ($E = V_{DS} / L$, where L is the channel length). A large multiplication factor of up to 5,000 was observed before permanent breakdown occurred, confirming that the impact ionization process generated a large number of carriers. The breakdown voltage depends strongly on the channel length and thickness of WSe_2. The channel length dependence on the breakdown voltage is shown in Supplementary Fig. 4d. To obtain the length dependence of the multilayer WSe_2 FETs, we used the same conditions for all devices (the large WSe_2 flake was divided into flakes of different lengths via etching). The breakdown voltage increases linearly with channel length, indicating that the critical electric field corresponding to the breakdown voltage is independent of channel length. We obtained an E_{CR} of approximately 30 kV/cm for the multilayer WSe_2 FETs. The thickness dependence of the critical electric field was also obtained, as shown in Supplementary Fig. 4e. The calculated E_{CR}s were 51.2, 41.2, 37.6, and 31.5 kV/cm for mono-, bi-, tri-, and multi-layer WSe_2, respectively. The field strength increased as the thickness decreased and was approximately related to the bandgaps of the samples. Supplementary Figure 4e also shows the thickness-dependent multiplication factors (shown with blue dots) measured at the same electric field (E = 52 kV/cm), for comparison. In Supplementary Fig. 4f, I_{DS} normalized by the saturation current is presented as a function of the electric field for various temperatures at $V_{GS(B)} = -30$ V.

c. Gate bias effect on impact ionization

Supplementary Figure 5 presents the gate bias effect on the abrupt rise of the drain current caused by impact ionization in the WSe_2 channel. Supplementary Figure 5a presents the I_{DS}-V_{DS} characteristics of the WSe_2 FET measured at various gate voltages ($V_{GS(B)}$). $V_{GS(B)}$ increases from −30 to −15 V, which is a charge neutral point for the target device. We observed that the avalanche breakdown voltage (V_{BR}) shifted into large magnitude according to changes in the gate bias (black symbols in Supplementary Figure 5b). The observed increase in V_{BR} with an increasing accumulated carrier concentration (blue symbols in Supplementary Figure 5b) can be attributed to enhanced carrier scattering in the WSe_2 channel. The carrier concentration was calculated using the formula $n = \frac{Q}{e} = \frac{C_{ox} \times (V_{GS} - V_{CNP})}{e}$, where C_{ox} is the capacitance of the SiO_2 dielectric layer, V_{CNP} is the charge neutral point, and e is the elementary charge.

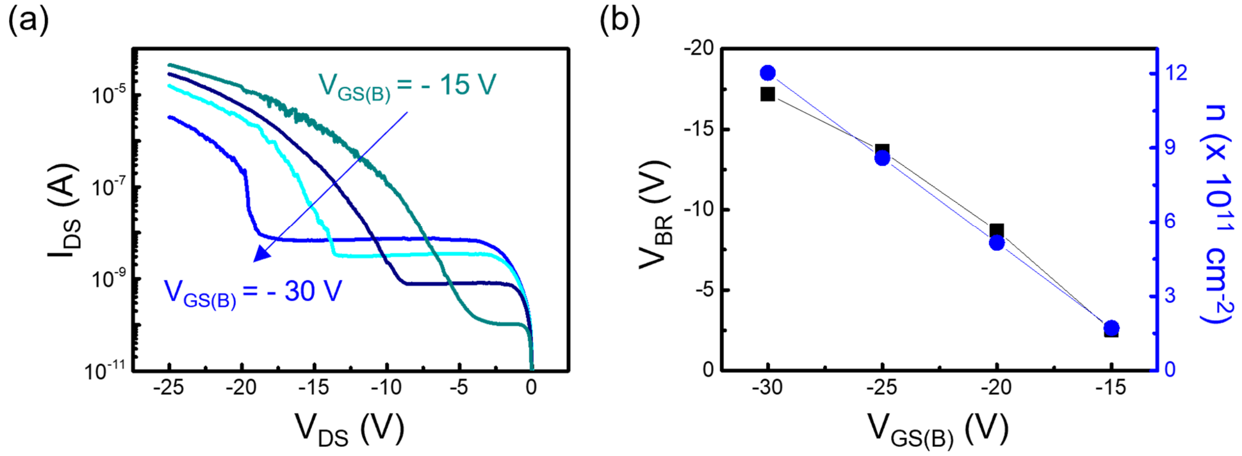

Supplementary Figure 5. (a) I_{DS}-V_{DS} characteristics for WSe_2 FET at various back gate voltages ranging from −15 to −30 V with a step of 5 V. (b) V_{BR} at which impact ionization initiates and the corresponding carrier concentration as a function of the back gate voltages.

d. Output characteristics during V_{DS} sweeps

Supplementary Figure 6 presents the output characteristics of WSe_2 FETs. The reversible output curves, including the impact ionization process, were measured under multiple V_{DS} sweeps. These results suggest that the WSe_2 channel was not damaged after the reversible impact ionization caused by thermal stress originating from Joule heating [3].

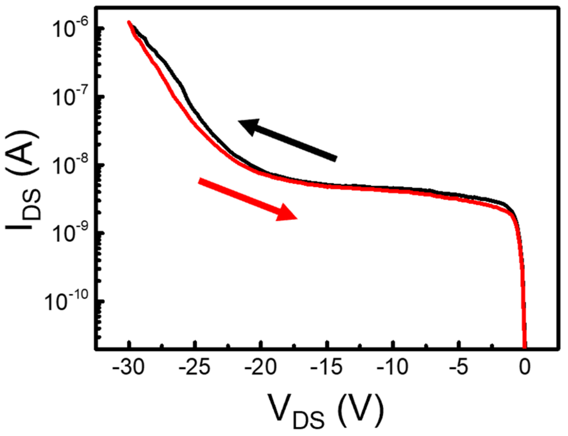

Supplementary Figure 6. I_{DS}-V_{DS} output characteristics of WSe_2 FETs. Sweep directions are marked by arrows.

e. Estimation of ionization coefficient (lucky drift mechanism)

The ionization coefficient based on the lucky drift mechanism [4] is given by

$$\alpha = \frac{e}{E_I} P(\xi, E_I), \tag{1}$$

where ξ is the strength of the applied electric field, E_I is the threshold energy required for impact ionization, and P (ξ, E_I) is the probability of avoiding a collision before reaching the threshold energy. There are two main modes in the lucky drift mechanism as follows: (i) lucky ballistic mode, where the carrier reaches the threshold without a momentum-relaxing collision ($0 < t \leq \tau_m$), and (ii) lucky-drift mode, where the carrier reaches the threshold without an energy-relaxing collision ($\tau_m \leq t \leq \tau_E$). Therefore, we have P (ξ, E_I) = P_1 (ξ, E_I) + P_2 (ξ, E_I). P_1 (ξ, E_I) and P_2 (ξ, E_I) represent the lucky ballistic and lucky drift modes, respectively, and are given by

$$P_1(\xi, E_I) = \exp\left(-\int_0^t \frac{dt}{\tau_m}\right) = \exp\left(-\int_0^{E_I} \frac{dE}{e\xi v_g \tau_m}\right), \tag{2-1}$$

$$P_2(\xi, E_I) = \exp\left(-\int_0^t \frac{dt}{\tau_E}\right) = \exp\left(-\int_0^{E_I} \frac{m^* dE}{e^2 \xi^2 \tau_E \tau_m}\right). \tag{2-2}$$

The mean free path was defined as λ= $v_g \tau_m$. To calculate the impact ionization coefficient of WSe_2, we considered the isotropic and parabolic energy bands of WSe_2. The dominant scattering mechanism at room temperature is due to relatively high-energy optical phonons

generated by the deformation-potential interaction. Polar optical phonon scattering can be ignored because it is weak at high energies. In such a case, the relationship between the momentum and energy relaxation times can be written as

$$\frac{\tau_E}{\tau_m} = \frac{2n(\omega)+1}{h\omega}E = \frac{E}{rE_I}, \tag{3}$$

where $n(\omega)$ is the Bose-Einstein number, $h\omega$ is the optical phonon energy of WSe_2, and r can be derived as

$$r = \frac{h\omega}{[2n(\omega)+1]E_I}. \tag{4}$$

For bulk tungsten diselenide with $E_I \approx 1.5E_{bandgap} = 1.5$ eV and $h\omega \approx 31$ meV, we can obtain that $r \approx 0.01$ at room temperature. Additionally, the mean free path λ is constant and independent of the energy, so we have

$$\frac{m^*}{\tau_m \tau_E} = \frac{2rE_I}{\lambda^2}. \tag{5}$$

With these parameters, we can obtain

$$\alpha = \frac{1}{x}\left\{e^{-x} + \left(\frac{e^{-2rx^2} - e^{-x}}{1-2rx}\right) + P_T\left[e^{-x(1-\sigma)} + \left(\frac{e^{-2rx^2(1-\sigma)} - e^{-x(1-\sigma)}}{1-2rx}\right)\right]\right\}, \tag{6}$$

with $= E_I/e\xi\lambda$, $\sigma = P_T/2rx^2$, and $P_T = 1 - e^{-2rx(x-3)}$.

The analytical expression of Eq. (6) can be extended to cover the scenario carrier injection at energy above zero. In this case,

$$\alpha\lambda = \frac{1}{x(1-\eta)}\left\{e^{-x(1-\eta)} + \left(\frac{e^{-2rx^2(1-\eta)}-e^{-x(1-\eta)}}{1-2rx}\right) + \frac{P_T}{x}\left[e^{-x(1-\sigma)} + \left(\frac{e^{-2rx^2(1-\sigma)}-e^{-x(1-\sigma)}}{1-2rx}\right)\right]\right\}, \tag{7}$$

where $\eta = E_0 / E_I$ and E_0 is the injection energy of the carrier. The thermalization component remains the same as in Eq. (6).

According to Eq. (7), the figure below presents the effects of injection energy on the ionization coefficient of bulk WSe_2 at room temperature. As η increases from 0 to 0.9, the ionization coefficient increases significantly for the same x, particularly at large values of η. This can be explained by Eq. (7) because the injected carrier energy E_0 reduces the distance to reach the threshold. However, we cannot achieve η=1 because it is physically impossible.

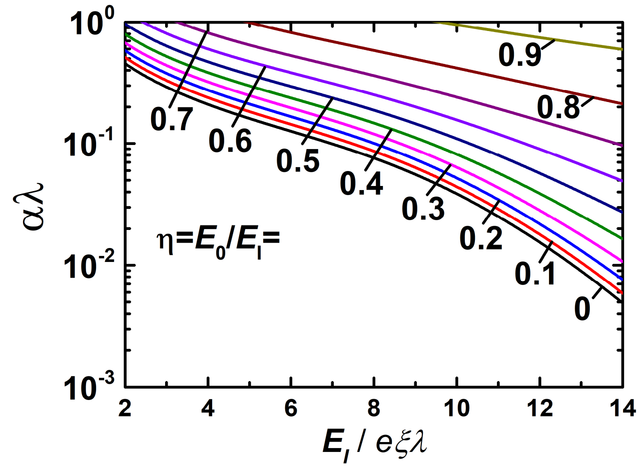

Supplementary Figure 7. The ionization coefficients of bulk WSe_2 at room temperature under various carrier injection energies E_0 starting from zero to $0.9E_I$, where E_I =1.5 eV.

f. Relationship between the multiplication factor and channel length

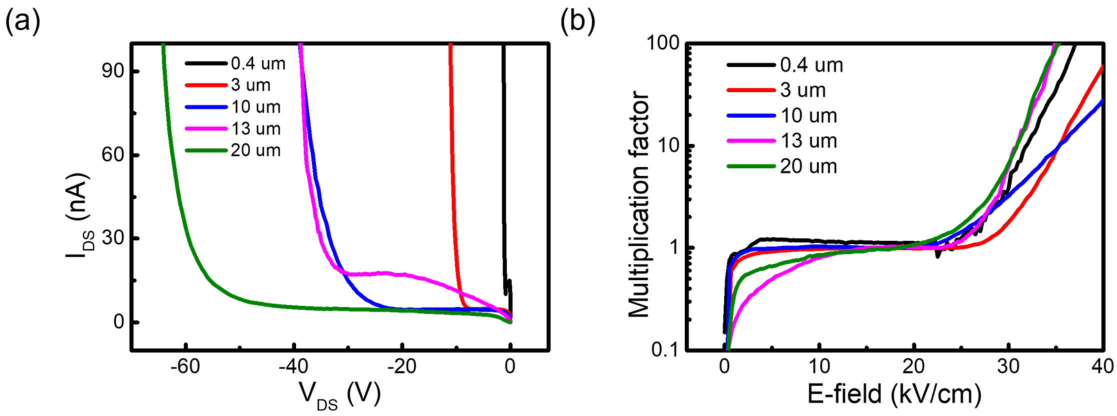

Supplementary Figure 8. (a) Output curves for various WSe_2 FETs with different channel lengths. (b) Multiplication factor versus applied electric field corresponding to (a).

Supplementary Figure 8 shows the channel length-dependent impact ionization characteristics. As shown in Supplementary Fig. 8a, the V_{BR} decreases linearly as the channel length decreases. It is determined by E_{CR}, which is an intrinsic property of the material, and follows the relationship of $V_{BR} = E_{CR} \times L$, where L is the channel length. To investigate the relationship between the multiplication factor (M) and channel length, the M values of several WSe_2 FETs with different channel lengths were plotted against the applied electric field, as shown in Supplementary Fig. 8b. When an electric field larger than E_{CR} is applied, all M values increase with a similar slope, indicating that M is independent of the channel length.

Supplementary Note 3. Properties and control of the WSe_2 I^2FET

a. Electrical properties of the WSe_2 I^2FET

The total channel resistance in the WSe_2 I^2FET consists of the gated region resistance (R_{gated}) and ungated region resistance ($R_{ungated}$) as $R_{total} = R_{gated} + R_{ungated}$, as shown in Supplementary Fig. 9a. The transfer curves and calculated resistivity of the WSe_2 FET as a function of only the back-gate bias $V_{GS(B)}$ and top-gate bias $V_{GS(T)}$ (at the charge neutral point, $V_{GS(B)} = -15$ V) are presented in Supplementary Figs. 9b and 9c, respectively. We calculated the resistance of the gated region and ungated region at the threshold voltage just before impact ionization occurred. The electrical parameters for WSe_2 I^2FETs with different gated region lengths (L_{gated}) and ungated region lengths ($L_{ungated}$) were calculated, as shown in Supplementary Table 1.

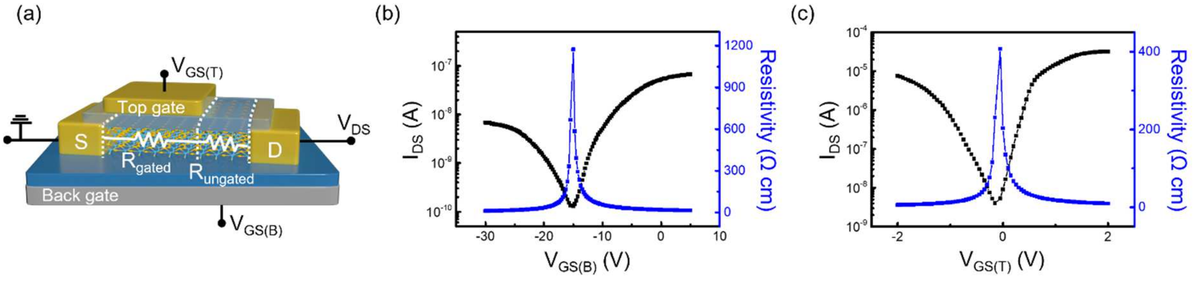

Supplementary Figure 9. (a) Schematic of the WSe_2 I^2FET with resistance components. Transfer characteristics (black symbols and line) and resistivity (blue symbols and line) with respect to (b) back-gate bias and (c) top-gate bias (with a fully covered top gate and back gate).

The resistivities of the gated region (ρ_{gated}) and ungated region ($\rho_{ungated}$) were extracted at the threshold voltage (V_{TH}) and charge-neutral point (V_{CNP}), respectively, as follows:

$$\rho = \frac{1}{\sigma} = \frac{1}{ne\mu},$$

where the carrier concentration was calculated as $n = \frac{Q}{e} = \frac{C_{ox} \times (V_{GS} - V_{CNP})}{e}$ (C_{ox} is the capacitance of each SiO_2 dielectric layer, V_{CNP} is the charge neutral point, and e is the elementary charge).

$$\rho_{gated} = \rho\left(@V_{GS(T)} = V_{TH}\right), \qquad \rho_{ungated} = \rho\left(@V_{GS(B)} = V_{CNP}\right)$$

The resistance of each region was calculated using the following equations, where W and t are the width and thickness of the channel, respectively:

$$R_{gated} = \rho_{gated} \times \frac{L_{gated}}{W \times t},\ R_{ungated} = \rho_{ungated} \times \frac{L_{ungated}}{W \times t}.$$

Through these calculations, we can estimate the voltage drop occurring within the I^2FET and the critical electric field in the ungated region, where impact ionization occurs.

$$V_{ungated} = V_{DS} \times \frac{R_{ungated}}{R_{ungated} + R_{gated}}, \qquad E_{CR} = \frac{V_{ungated}}{L_{ungated}}$$

Gated-region				Ungated-region				V_{DS} (V)	$V_{ungated}$ (V)	E_{CR} (kV/cm)
L_{gated} (μm)	V_{TH} (V)	ρ_{gated} (Ω cm)	R_{gated} (MΩ)	$L_{ungated}$ (nm)	V_{CNP} (V)	$P_{ungated}$ (Ω cm)	$R_{ungated}$ (MΩ)			
5	−1.09	11.6	4.64	300	−15	667	16	3	2.33	77.5
3	−0.84	15.4	3.70	330	−15	667	17.6	3	2.48	75.1
2	−0.48	28.9	4.62	300	−15	667	16	3	2.33	77.6
1	−0.42	38.5	3.08	350	−15	667	18.7	3	2.58	73.6
5	−1	12.8	5.12	300	−15	667	16	3	2.27	75.8
3	−1	12.8	3.07	330	−15	667	17.6	2.9	2.47	74.8
2	−1	12.8	2.05	300	−15	667	16	2.6	2.31	76.8
1	−1	12.8	1.02	350	−15	667	18.7	2.8	2.65	75.8

Supplementary Table 1. Summarized resistance values and calculated voltage drops for several WSe_2 I^2FETs fabricated from the same flake (width: W_{gated} = $W_{ungated}$ = 2.5 μm, thickness: t_{gated} = $t_{ungated}$ = 50 nm).

The calculated critical electric field values are very similar, but slightly higher than the WSe_2 impact ionization properties investigated. This can be attributed to the simplified calculation of the carrier concentration and contact resistance.

b. Control of operating voltages via gated and ungated region length modulation

By adjusting the device dimensions (gated: gated region length, ungated: ungated region length) of the WSe_2 I^2FET, the two critical biases can be controlled and scaled down. These are the magnitude of the drain voltage (V_{BR}) at which impact ionization induces a sudden increase in drain current and the magnitude of the gate threshold voltage (V_{TH}) at which a steep transition of drain current from the off to the on state occurs. The control of V_{BR} and V_{TH} is critical because the eventual operating voltages of devices is determined by these parameters.

Considering that the main mechanism of our I^2FET is based on impact ionization in the ungated region, which is controlled by the amount of electric field applied in $L_{ungated}$, we expect to control V_{BR} and V_{TH} via the modulation of L_{gated} and $L_{ungated}$, and provide potential guidelines for the further scaling of the operating voltage. Supplementary Figures 10b and 10c present the output and transfer characteristics of the WSe_2 I^2FET with a fixed gated region length of approximately 3 μm with respect to different ungated region lengths ($L_{ungated}$ = 70, 130, 180, and 300 nm). As shown in Supplementary Fig. 10a, as $L_{ungated}$ decreases, V_{BR} and V_{TH} decrease monotonically because a reduced voltage is required to reach the critical electric field E_{CR} with a shorter $L_{ungated}$. These results demonstrate that scaling V_{DS} to less than 1 V and $V_{GS(T)}$ to less than 0.5 V can be achieved with $L_{ungated}$ = 70 nm, suggesting that further scaling of operating voltages can be achieved by utilizing commercial lithography and patterning capabilities. Similar experiments and analyses were conducted with a fixed ungated region length of approximately 330 nm while varying the gated region length (L_{gated} = 1, 2, 3, and 5 μm). Supplementary Figure 10d presents the transfer characteristics of these devices. As expected, the scaling down of V_{BR} and V_{TH} was achieved by decreasing L_{gated} (Supplementary Figure 10e). In summary, we can reduce the breakdown voltage by scaling down the gated region or ungated region length, as shown in Supplementary Fig. 10f.

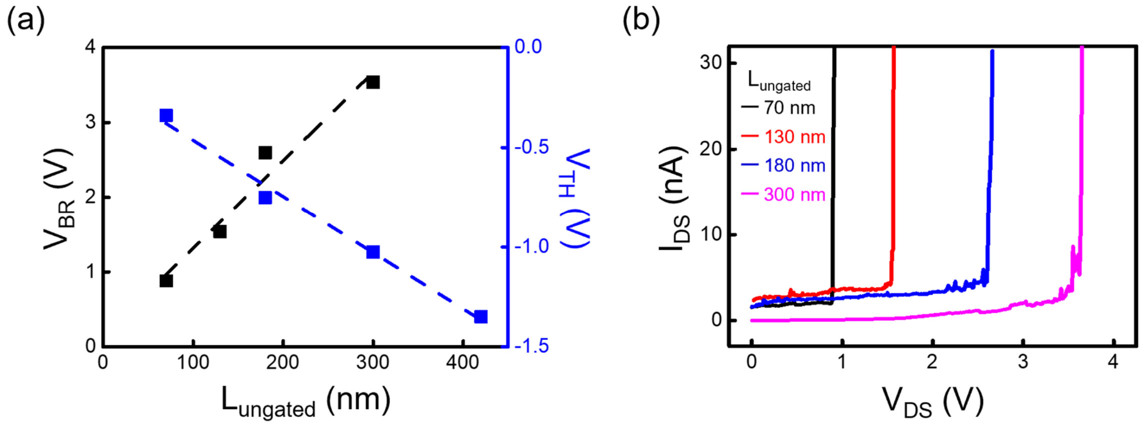

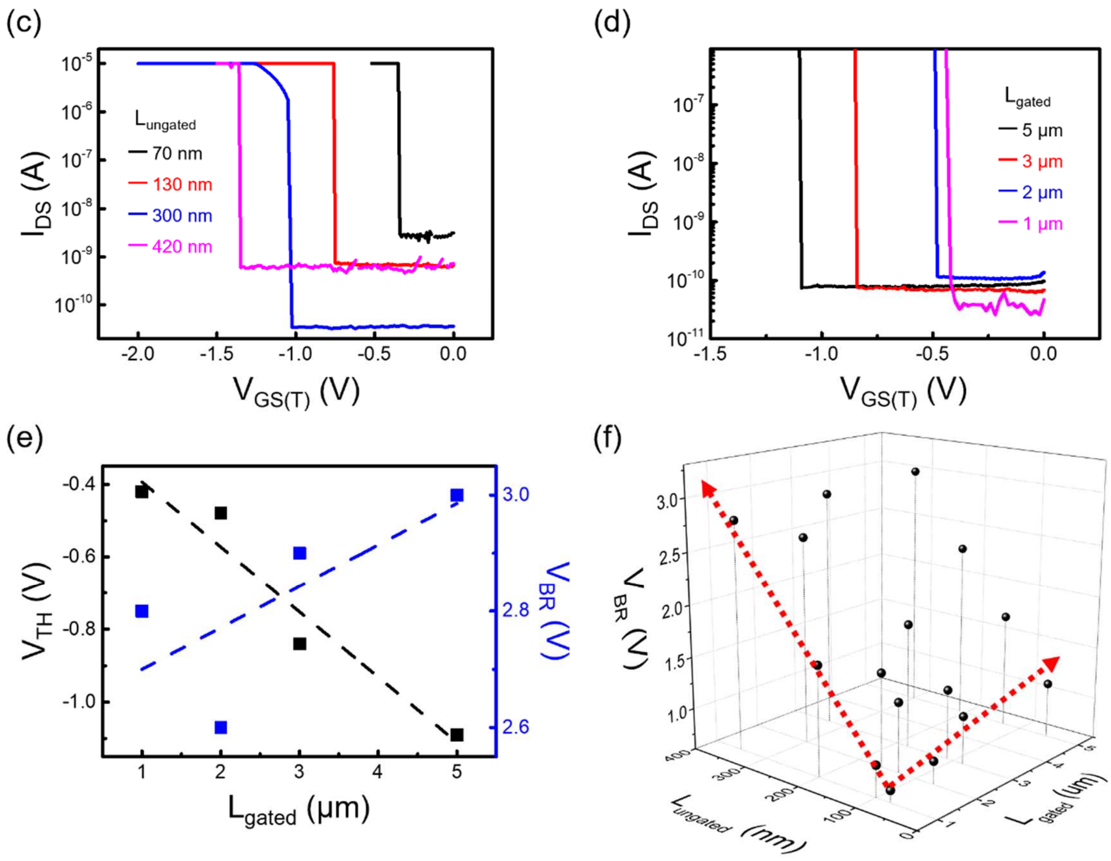

Supplementary Figure 10. (a) Ungated region dependency of V_{BR} and V_{TH}. (b) Output curve characteristics (at the same $V_{GS(T)} = -3$ V) and (c) Transfer curve characteristics (at same $V_{DS} = 3$ V) of the WSe_2 I^2FET with different ungated region lengths ($L_{ungated}$). As $L_{ungated}$ decreases, the magnitudes of both V_{BR} and V_{TH} decrease. (d) I_{DS}-$V_{GS(T)}$ characteristics with different gated region lengths and the same ungated region length. (e) Scaling of V_{TH} and V_{BR} with a decreasing gated region length. (f) V_{BR} values (at $V_{TH} = -3$ V) of different I^2FETs with various ungated and gated region lengths.

c. Negligible hysteresis characteristics of the impact ionization process

Supplementary Figure 11 presents the drain current as a function of the top-gate bias, which was swept in opposite directions. A small hysteresis in the drain current can be observed (inset in Supplementary Fig. 11). This is mainly caused by the fact that the proposed device is composed of a homogeneous WSe_2 junction, unlike previously reported heterostructures [5, 6] stacked with several different layers, where the introduction of trap sites is unavoidable. In addition, this little hysteresis supports that our device is based on an impact ionization

mechanism rather than a feedback mechanism.

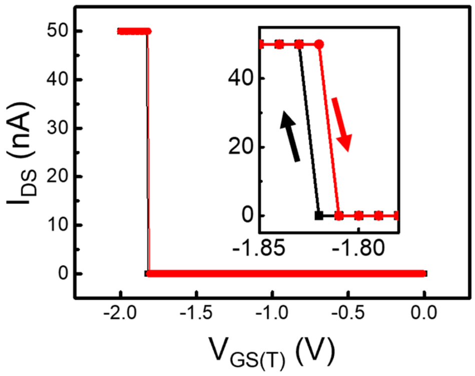

Supplementary Figure 11. Hysteresis curves of WSe_2 I^2FETs. (Inset: magnification of the hysteresis curves). Sweep directions are marked by arrows.

d. Gate leakage current

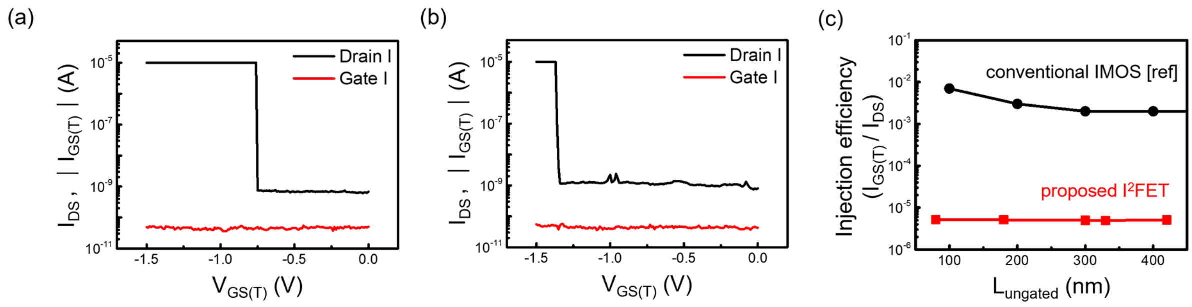

Supplementary Figure 12. Drain current and gate leakage current versus top-gate voltages of WSe_2 I^2FETs with (a) $L_{ungated}$ = 180 nm and (b) $L_{ungated}$ = 420 nm with V_{DS} = 3 V. The gate oxide leakage current has an insignificant value compared to the drain current. (c) Calculated injection efficiency for various devices with different $L_{ungated}$s compared with conventional IMOS.

Supplementary Figures 12a and 12b compare the drain and gate leakage currents of WSe_2 I^2FETs with different channel lengths, respectively. A significant level of gate leakage current generally indicates poor reliability because although a certain number of carriers make it through the barrier, there are others that get trapped in the oxide or create interface states, causing damage [7]. Supplementary Figure 12c exhibits the injection efficiency, defined as the ratio of the gate current to the drain current (I_{GS}/I_{DS}). In contrast to the conventional I-MOS, our device shows a significantly lower injection efficiency, indicating a low hot-carrier trapping rate. Our WSe_2 I^2FET shows a low gate leakage current (<0.1 nA with a low injection efficiency $<10^{-5}$).

e. Reliability of the impact ionization phenomenon

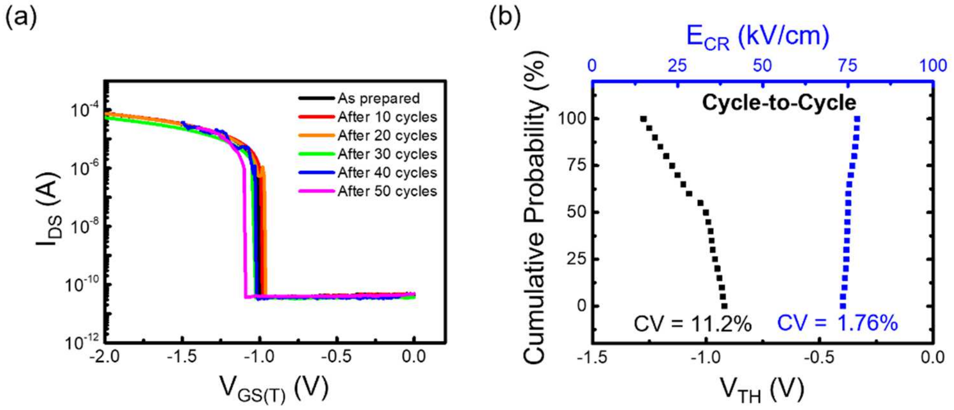

Supplementary Figure 13. (a) Repeated measurement results recorded every 10 cycles. (b) V_{TH} (black dots) and E_{CR} (blue dots) during repeated steep switching phenomena are shown as cumulative probability curves.

Supplementary Figure 13b illustrates the cumulative probabilities of the threshold voltage (V_{TH}) and critical electric field (E_{CR}) obtained from cycle-to-cycle data (results from the same device over several cycles) over 50 cycles, as shown in Supplementary Fig. 13a. After tens of cycles, similar impact ionization characteristics were observed, indicating that no apparent degradation occurred except for slight changes in the breakdown voltage and current multiplication. Reliability was quantified using the coefficient of variation (CV). CV is commonly used to measure the dispersion of probability distributions and can be calculated using the following expression: $CV = (\sigma/\mu) \times 100\ (\%)$, where σ is the standard deviation, and μ is the absolute mean value.

We believe that the repeatable operation of the fabricated impact-ionization FET can be attributed to factors such as the greatly reduced V_{ds} and highly asymmetric transport properties of 2D materials, making carriers flow parallel to the plane.

First, the previously studied materials usually require a high critical electric field (E_{CR}, usually over 300 kV/cm) to trigger impact ionization. By contrast, we found that WSe_2 has a low E_{CR} of 20–50 kV/cm, which is a very meaningful finding, indicating that a greatly reduced drain voltage can trigger impact ionization in our device. We need to apply a voltage of 0.6–1.5 V for our WSe_2 I^2FET to generate impact ionization in the ungated intrinsic region. With this scaled bias condition, the energy and number of hot carriers will be greatly reduced; therefore, the hot-carrier-induced reliability concern can be addressed.

Second, in conventional bulk materials used in IMOS, such as Si, a major current flow occurs near the interface in contact with the dielectric, where charges are created by gate-voltage modulation. However, in two-dimensional layered materials such as WSe_2, the location of the "HOT-SPOT," where the current mainly flows, is determined by the gate voltage modulation and number of layers [8]. For a two-dimensional layered system with multiple layers, as the gate voltage increases from the threshold voltage, the HOT-SPOT is located further away from the dielectric. It is expected that the position of the HOT-SPOT changes approximately 20–24 layers below the dielectric, which can be significant in suppressing the gate leakage current (as can be seen in Supplementary Fig. 12) and hot-carrier-induced damage to the dielectric.

Supplementary Note 4. Complementary inverter with WSe_2 I^2FET

a. Noise margins of WSe_2 I^2FET inverter

The noise margin (NM) is another important parameter in the context of the inverter and determines the stability of the output with respect to the signal interference at the input. As shown in Fig. 5d, the input voltage is at logic high ("1") if $V_{IN} < V_{IH}$ and at logic low ("0") if $V_{IN} > V_{IL}$. This is because the input voltages in these ranges can produce unambiguous output logic levels. V_{OH} and V_{OL} are the ideal logic high and logic low of the inverter, respectively, as shown in Supplementary Fig. 14. The high noise margin (NM_H) and low noise margin (NM_L) are then calculated according to the expressions $NM_H = V_{OH} - V_{IH}$ and $NM_L = V_{IL} - V_{OL}$ as $NM_H = 0.985$ V = 98.5% of $1/2V_{DD}$ and $NM_L = 1.012$ V = 101.2% of $1/2V_{DD}$ for $V_{DD} = 2$ V. Both noise margins approach the ideal noise margin ($1/2V_{DD}$), indicating that the WSe_2 I^2FET inverter is robust to electrical noise in the environment and suitable for integration into multi-stage logic circuits.

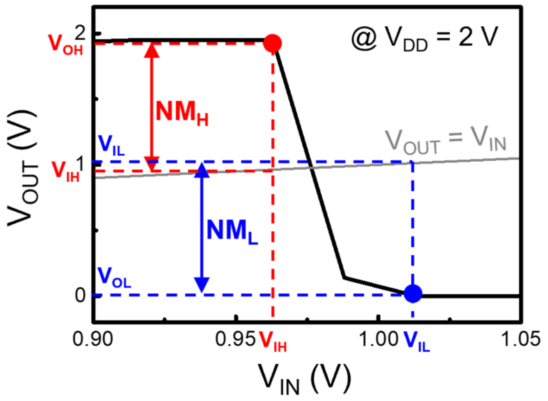

Supplementary Figure 14. Magnified view of the voltage transfer curve of the WSe_2 I^2FET inverter showing the input low and high (V_{IL} and V_{IH}) and output low and high (V_{OL} and V_{OH}) at $V_{DD} = 2$ V.

b. Scalable voltage drop of WSe_2 I^2FET inverter

Because our WSe_2 I^2FET is based on an impact ionization mechanism, there is a voltage drop over the region where the impact ionization occurs. However, this voltage drop follows the relationship of $V_{BR} = E_{CR} \times L$, where L is the channel length, and can be reduced by reducing the channel length. Supplementary Figure 15a shows the VTC curve of an inverter composed

of an I^2FET device with an ungated region length of approximately 300 nm. At this time, a voltage drop of approximately 0.7 V, similar to the theoretically expected voltage drop of 20 kV/cm × 300 nm = 0.6 V, was experimentally measured. The voltage drops in inverters composed of different I^2FETs with various ungated region lengths are summarized in Supplementary Fig. 15b. The blue dashed line indicates the expected ideal voltage drop calculated from the investigated E_{CR} of 20 kV/cm, and the experimental values indicated by the black squares follow the same trend.

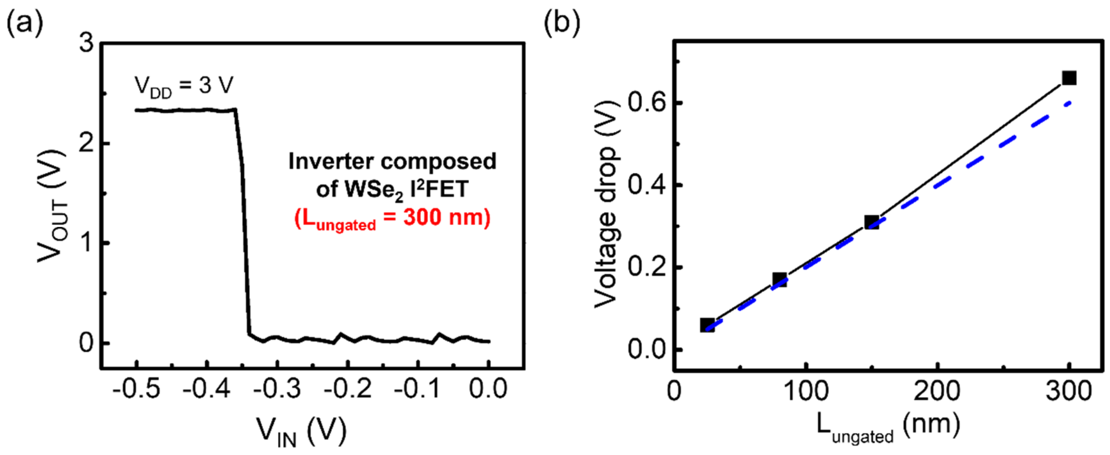

Supplementary Figure 15. (a) Inverter characteristics based on the WSe_2 I^2FET (with ungated region length of 300 nm) in series with n-MoS_2 FET. (b) Calculated expected voltage drop (blue dashed line) and experimentally measured voltage drops (black squares) with various ungated region lengths.

Device type	Material	V_{DD} (V)	Peak gain	$(NM_L+NM_H)/V_{DD}$	SS (mV/dec)	Ref
TFET	Si NW	1.2	58	N/A	30	[9]
	Si	1	18	N/A	42	[10]
	Ge/InAs	1	30	N/A	55	[11]
NC-FET	MoS_2/WSe_2	2	12	0.9	31	[12]
	In_2O_3 NW	5	41.6	N/A	10	[13]
	MoS_2/CIPS	1.5	23	0.899	28	[14]
MEMS	BCB (Polymer)	2	80	N/A	N/A	[15]
I-MOS	Si	1	45.7	0.593	8.9	[16]
This Work	WSe_2/MoS_2	2	72.8	0.999	2.73	-

Supplementary Table 2. Comparison between complementary logic inverters based on various steep switching devices. Our inverter demonstrates the highest peak gain and noise margin.

Supplementary References

[1] Giannozzi P, *et al.* QUANTUM ESPRESSO: a modular and open-source software project for quantum simulations of materials. *J. phys. Condens. matter* **21**, 395502 (2009).

[2] Grimme S, Antony J, Ehrlich S, Krieg H. A consistent and accurate ab initio parametrization of density functional dispersion correction (DFT-D) for the 94 elements H-Pu. *J. chem. phys.* **132**, 154104 (2010).

[3] Behranginia A, *et al.* Power Dissipation of WSe_2 Field-Effect Transistors Probed by Low-Frequency Raman Thermometry. *ACS Appl. Mater. Interfaces* **10**, 24892-24898 (2018).

[4] Ridley B. Lucky-drift mechanism for impact ionisation in semiconductors. *J. Phys. C: Solid State Phys.* **16**, 3373 (1983).

[5] Li M-Y, Chen C-H, Shi Y, Li L-J. Heterostructures based on two-dimensional layered materials and their potential applications. *Mater. Today* **19**, 322-335 (2016).

[6] Dong R, Kuljanishvili I. Review Article: Progress in fabrication of transition metal dichalcogenides heterostructure systems. *J. Vac. Sci. Technol. B Nanotechnol. Microelectron.* **35**, 030803 (2017).

[7] Mayer F, Le Royer C, Blachier D, Clavelier L, Deleonibus S. Avalanche breakdown due to 3-D effects in the impact-ionization MOS (I-MOS) on SOI: reliability issues. *IEEE Trans. Electron Devices.* 55, 1373-1378 (2008).

[8] Das S, Appenzeller J. Where does the current flow in two-dimensional layered systems? *Nano Lett.* 13, 3396-3402 (2013).

[9] Knoll L, *et al.* Inverters With Strained Si Nanowire Complementary Tunnel Field-Effect Transistors. *IEEE Electron Device Lett.* **34**, 813-815 (2013).

[10] Ionescu AM, Riel H. Tunnel field-effect transistors as energy-efficient electronic switches. *Nature* **479**, 329-337 (2011).

[11] Ionescu AM, *et al.* Ultra low power: Emerging devices and their benefits for integrated circuits. In: *2011 International Electron Devices Meeting (IEDM*). 16.1.1-16.1.4 (IEEE, 2011).

[12] Wang J, *et al.* Low-Power Complementary Inverter with Negative Capacitance 2D Semiconductor Transistors. *Adv. Funct. Mater.* **30**, 2003859 (2020).

[13] Xu Q, *et al.* In_2O_3 Nanowire Field-Effect Transistors with Sub-60 mV/dec Subthreshold Swing Stemming from Negative Capacitance and Their Logic Applications. *ACS Nano* **12**, 9608-9616 (2018).

[14] Wang X, *et al.* Van der Waals negative capacitance transistors. *Nat. Commun.* **10**, 3037 (2019).

[15] Song YH, *et al.* High-performance hybrid complementary logic inverter through monolithic integration of a MEMS switch and an oxide TFT. *Small* **11**, 1390-1395 (2015).

[16] Choi WY. Applications of impact-ionization metal–oxide-semiconductor (I-MOS) devices to circuit design. *Curr. Appl. Phys.* **10**, 444-451 (2010).
